# Mobile Sensing with Smart Wearables of the Physical Context of Distance Learning Students to Consider Its Effects on Learning

**DOI:** 10.3390/s21196649

**Published:** 2021-10-07

**Authors:** George-Petru Ciordas-Hertel, Sebastian Rödling, Jan Schneider, Daniele Di Mitri, Joshua Weidlich, Hendrik Drachsler

**Affiliations:** 1 Educational Technologies, Information Center for Education, DIPF|Leibniz Institute for Research and Information in Education, 60323 Frankfurt am Main, Germany; roedling@dipf.de (S.R.); schneider.jan@dipf.de (J.S.); dimitri@dipf.de (D.D.M.); weidlich@dipf.de (J.W.); drachsler@dipf.de (H.D.); 2 Computer Science Faculty, Goethe University, 60323 Frankfurt am Main, Germany; 3 Educational Science Faculty, Open University of the Netherlands, 6419 AT Heerlen, The Netherlands

**Keywords:** multimodal learning analytics, physical learning environment, learning context, mobile sensing, self-directed learning, distance learning, smart wearables, software infrastructure, Edutex

## Abstract

Research shows that various contextual factors can have an impact on learning. Some of these factors can originate from the physical learning environment (PLE) in this regard. When learning from home, learners have to organize their PLE by themselves. This paper is concerned with identifying, measuring, and collecting factors from the PLE that may affect learning using mobile sensing. More specifically, this paper first investigates which factors from the PLE can affect distance learning. The results identify nine types of factors from the PLE associated with cognitive, physiological, and affective effects on learning. Subsequently, this paper examines which instruments can be used to measure the investigated factors. The results highlight several methods involving smart wearables (SWs) to measure these factors from PLEs successfully. Third, this paper explores how software infrastructure can be designed to measure, collect, and process the identified multimodal data from and about the PLE by utilizing mobile sensing. The design and implementation of the Edutex software infrastructure described in this paper will enable learning analytics stakeholders to use data from and about the learners’ physical contexts. Edutex achieves this by utilizing sensor data from smartphones and smartwatches, in addition to response data from experience samples and questionnaires from learners’ smartwatches. Finally, this paper evaluates to what extent the developed infrastructure can provide relevant information about the learning context in a field study with 10 participants. The evaluation demonstrates how the software infrastructure can contextualize multimodal sensor data, such as lighting, ambient noise, and location, with user responses in a reliable, efficient, and protected manner.

## 1. Introduction

In times of a pandemic, students are encouraged to keep their physical distance from each other and study from home if possible. In this scenario, distance learning becomes essential in areas where it was not before, and more institutions increase their efforts to digitize their teaching materials and structure their learning courses in learning management systems. Understanding how students use learning materials and digital learning environments can be beneficial to collect data about learners’ behavior. This approach is most commonly referred to as Learning Analytics (LA). The Society for Learning Analytics Research (SoLAR) defined Learning Analytics as the measurement, collection, analysis, and reporting of data about learners and their contexts for purposes of understanding and optimizing learning and the environments in which it occurs (https://www.solaresearch.org/about/what-is-learning-analytics/, last accessed on 9 February 2021). Traditionally, LA research focuses on learner behavior as it interfaces with the keyboard and mouse in the learning management system. This narrow perspective on learner behavior in digital environments can lead to incomplete or ambiguous data traces because many other factors are difficult to capture and thus cannot be taken into account [1]. To counteract such potential shortcomings, approaches such as multimodal learning analytics (MMLA) are used [2]. MMLA has been used to gather rich data on various learning tasks, such as collaboration (e.g., [3]), public speaking (e.g., [4,5]), or CPR training (e.g., [6]). In addition to supporting conscious behavioral activities, MMLA can also be used to collect and process physiological (e.g., [7]) and contextual data (e.g., [8]).

**Figure 1:**
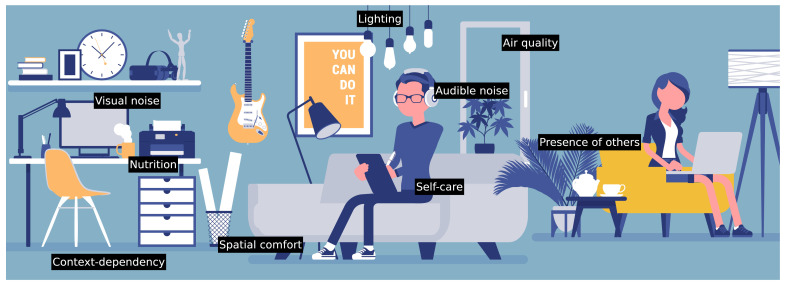
This figure illustrates an exemplary learning space with nine flagged factors that can effect learning. The factors identified through a literature search and flagged here are visual noise, audible noise, context dependency, air quality, nutrition, lighting, spacial comfort, self-care, and presence of others. (From stock.adobe.com by andrew_rybalko).

Many factors might have a direct or indirect effect on learning, some of which may emanate from the physical learning environment (PLE) [9], such as lighting, temperature, or noise level. Figure 1 shows an example of a learning space with potentially affecting factors. Previous research has investigated the effects of physical environment factors on learners and has shown that the configuration of certain environmental factors can benefit or hinder performance in selected learning tasks [10,11]).

Various methods and instruments have been used for this purpose. One instrument which was selected for this purpose is mobile sensing. Mobile sensing is a form of passive natural observation of a participant’s daily life, using mobile sensor-equipped devices to obtain ecologically valid measurements of behavior. Mobile sensing often uses a variety of biometric sensors and data from self-reports utilizing, for example, the Ecological Momentary Assessment (EMA). Particular devices such as movisens (https://www.movisens.com/, last accessed on 5 August 2021) can be used as instruments. However, by using commodity devices such as smartphones and smartwatches that students already own, studies can reach more subjects and research prototypes can be more easily transformed into simple learning support tools. Such simple tools could support everyday learning by allowing students to journal their learning contexts and learning behaviors to reflect on them.

This paper is broadly concerned with how LA data can be augmented by considering the physical context of learners engaging in distance learning from home. Specifically, we investigate how multimodal data about the PLE with potential effects on learning can be measured, collected, and processed while utilizing mobile sensing with commodity hardware. For this reason, this paper presents Edutex, a software infrastructure that can leverage consumer smartwatches and smartphones for this purpose. Edutex is an implementation of the Trusted and Interoperable Infrastructure for Learning Analytics (TIILA) [12] with a specialization in mobile sensing through smart wearables.

The first step in achieving this goal is to identify the factors from the students’ PLE that might have an effect on their learning. Once identified, these factors need to be measured with adequate instruments. From these steps, we derive the following two research questions:

RQ1 Which factors of the physical learning environment can have an effect on distance learning?
RQ2 Which instruments can be used to measure factors from the physical learning environment?

Based on the foundation laid by answering research questions RQ1 and RQ2, we will derive requirements and design and implement the infrastructure. From the literature search needed to answer RQ1 and RQ2, we expect to learn, as described, which factors are already known and to what extent they can have an effect on learning. However, we still need to determine which data can be efficiently measured and collected and whether they can effectively describe the factors. By analyzing and aggregating the value set in terms of consistency and coherence, it should be possible to determine the physical context during specific learning periods. By deploying the development in a realistic pilot study, we will assess whether the prototype can thus provide potentially relevant information about the learners’ context in relation to the insight gained from the literature search. With these goals in mind, we formulate the following two research questions:

RQ3 How can software infrastructure be designed to measure, collect, and process multimodal data about the physical learning environment through mobile sensing?
RQ4 To what extent can the developed software infrastructure provide relevant information about the learning context in a field study?

The remainder of this paper is structured into five sections. In the following background section (Section 2), we address the background of our research by answering research questions RQ1 and RQ2. In said section, we explain the results of our literature search on factors from the PLE that might have an effect on learning (RQ1) and we review the instruments that have already been used to measure these factors in the literature (RQ2). In the software infrastructure section (Section 3), this paper describes the developed concepts. This description elaborates on the developed software design and the design considerations. In the method section (Section 4), we explain the study design and setup we used to answer our research questions. In the results section (Section 5), we illustrate the results we obtained from a study we conducted with ten participants. We then discuss these results in the discussion section (Section 6). There, we assess the quality of the data, the implementation’s performance, and the participants’ feedback. Finally, we draw a conclusion in Section 7.

## 2. Background

To measure relevant factors from a student’s PLE, they first need to be identified. Here, it is necessary to distinguish between factors from the physical environment and their effects on the student. After identifying the factors and effects, it is also desirable to determine how these identified factors can be measured with instruments in general and with smart wearable devices in particular.

**Figure 2:**
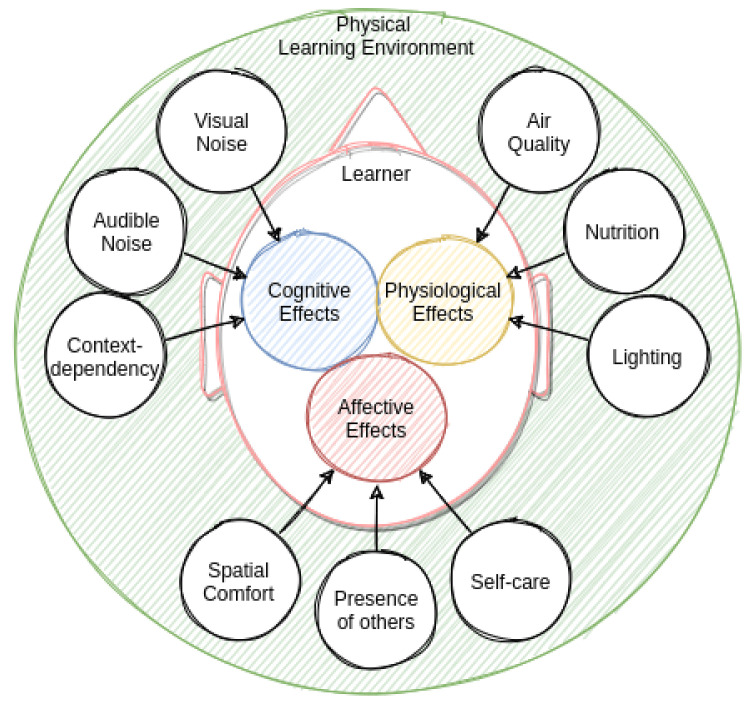
This illustration depicts the relationship between the nine identified factors from the physical learning environment and their effects on learners’ cognition, physiology, and affect.

Previous work has examined the relationship between the physical environment and various effects on learners’ cognitive loads (e.g., [9]). Similarly, we also attempted to assign categories to the effects we found in our literature search. In the three categories we chose, we distinguish between cognitive, physiological, and affective effects. Figure 2 visually summarizes the results of the literature search by depicting the factors from the PLE and their effects on the learner. In the following section, we describe these factors and their effects in detail.

### 2.1. Factors with Cognitive Effects

The factors of the physical environment that we have identified to have a cognitive effect on learning include visual and auditory noise, as well as context dependency. The cognitive effects relate to working memory and long-term memory.

#### 2.1.1. Visual Noise

Visual noise might be one of the most noticeable factors. It can be caused by everything from muted TVs or video streaming to people moving around in eyesight. Such visual noise can be regarded as an irrelevant environmental stimulus that drains the limited resources of working memory from learners’ cognitive processes (e.g., [13,14]). Studies have shown that cognitive performance can be improved simply by having subjects avert their gaze from their surroundings.

A way to measure visual noise is to capture and categorize browser content or smartphone usage (e.g., [15]). With the help of plugins and apps, smartphone app usage can be recorded and matched against classification lists. Nevertheless, even such lists can have difficulty distinguishing between learning usage and noise when it comes to YouTube videos or Facebook groups. Other visual noise, such as people interacting with each other [16] or with objects [17] in the environment, can be measured with 3D cameras.

#### 2.1.2. Auditory Noise

Auditory noise is another very noticeable factor. The sources of ambient noise and sounds can be multifaceted, for example, natural environmental sounds such as the wind blowing or birds chirping and conversations, music, or office noise. Several studies have examined the negative effects of machine noise, such as telephones ringing and conversations, on cognitive performance in work environments (e.g., [18]). The effect of ambient noise was also studied in a more detailed way in relation to learning. Irrelevant auditory sounds such as background speech or white noise were found to have a negative effect on learners’ working memory function (e.g., [19,20]).

Many smart devices already have a built-in microphone to record speech or voice commands. This integration makes it possible to use existing smart wearables or smart speakers for acoustic noise detection. When audio recording using smart wearables is carried out in an uncontrolled environment such as at home, the quality of accurate auditory detection of ambient noise can be questioned. A recent study found that the quality of smartwatch audio recordings is in principle sound enough for humans to recognize speech and other ambient sounds [21]. However, it was also found that more sophisticated voice activity detection tools are needed to accomplish this automatically. Smartphone microphones have already been used to measure and detect noise exposure [22]. However, it was noted that measurements may differ between different models due to the different sensors installed. Therefore, for fine measurements, it may be necessary to calculate a calibration offset for each device, complicating generic applications.

#### 2.1.3. Context Dependency

The contextual encoding of information in learning processes is another critical factor to consider. Memory performance is improved when the PLE and the physical test environment are similar, in contrast to when they are different (e.g., [23,24]). Contextual cues that the brain stores unconsciously and automatically about environmental stimuli from the PLE can be used to retrieve the same information later. Such contextual cues include olfactory cues such as smell [25], visual cues such as background color or visual markers [26], and auditory cues such as sounds and music [27]. When the context of a test situation cannot be mimicked, learning should best take place in various contexts, so that transfer to new contexts is facilitated [28].

With the help of location services, learners could become aware of which locations they use for learning, which ones they prefer, and with the additional use of assessments, perhaps even at which ones they seem to learn effectively. Outdoor location tracking is reasonably accurate and straightforward using geolocation services such as GPS. Additionally, some smartphone operating systems use Wi-Fi, Bluetooth, and cell towers to increase accuracy (e.g., [29]). These additional signals can also be leveraged to pinpoint locations in places where geolocation signals are not available, such as indoors. In addition, technologies such as RFID and NFC offer the ability to identify precise positions. Even room-level location in home environments is possible with smartwatches by generating activity fingerprints with data from a smartwatch’s microphone and inertial sensors and location information obtained from a smartphone [30].

### 2.2. Factors with Physiological Effects

Factors in the physical environment can also influence learning by affecting the learner’s physiology. The research literature reports three factors of interest in this regard. These three factors are air quality, nutrition, and lighting conditions. The effects found relate to learner willingness to exert effort, cognitive performance, and alertness.

#### 2.2.1. Air Quality

Cognitive performance is related to blood oxygen saturation (e.g., [31,32]). In this context, the temperature and oxygen level in the ambient air can be influencing factors. Learners may be more often aware of the relationship of their learning performance with the oxygen level of the ambient air than they are of the fact that a high ambient temperature can likewise reduce blood oxygen saturation and thus have an influence. For example, it has been shown that subjects working in an ambient temperature of 30 ${}^{\circ }$C show a lower willingness to exert effort than subjects working in an ambient temperature of 22 ${}^{\circ }$C [33].

The level of oxygen, CO${}_{2}$, and other volatiles in the air can be measured using special sensors [34]. Measuring the outside temperature is more challenging with smart wearables, as they are usually worn directly on the body and are thus affected by body temperature. Some unique smartphones use a laser sensor to measure the temperature of objects (https://www.catphones.com/de-de/cat-s61-smartphone, last accessed on 24 June 2021). However, most smart wearables can only measure their internal temperature. Nevertheless, this sensor and the small differences in relation to changing environments can be used to estimate the ambient temperature [35]. Sometimes, it might be easier to use public weather service data to estimate the temperature roughly. The most accurate method for this purpose would be to measure the blood oxygen saturation directly. It is possible with greater effort to use the cameras of ordinary smartphones with the help of dedicated models to measure oxygen saturation. Meanwhile, however, some smartwatches are already equipped with such a sensor, and the operating systems natively support them (https://www.apple.com/apple-watch-series-6/, last accessed on 5 August 2021).

#### 2.2.2. Nutrition

Learners’ nutrition may have an indirect effect on learning through the supply of energy to the brain. An elevated blood glucose level is associated with improvements in cognitive performance (e.g., [31]). Glucose, for example, is needed to meet the increased metabolic demands of the brain during demanding cognitive tasks. Not considering the health aspects, the consumption of glucose-intensive foods can increase learning performance for a short time [36].

Using standard sensors, such as an accelerometer, the eating behavior of a person can be detected reasonably accurately (e.g., [37]). Such a method could be used as a crude indicator without knowledge of the actual blood glucose effect. A more accurate method would be to directly measure the blood glucose concentration using, for example, sensors that evaluate the composition of a person’s sweat (e.g., [38]). However, the use of these sensors in smart wearables is still experimental (e.g., [39]).

#### 2.2.3. Lighting

The characteristics of light sources in a PLE can have different effects on learners. Here, the color and luminance of light sources can affect cognitive performance (e.g., [40,41]). Exposure to different wavelengths, for example, may have a positive effect on learners’ alertness and thus enhance their cognitive performances [42]. In addition, the level of concentration of learners could be improved with the help of color temperature and intensity [43,44].

A wide variety of devices already use light sensors to automatically adjust their screen’s illuminance and color temperature depending on the given lighting conditions. The objective of this functionality in smart wearables is typically to improve the visibility of the screen and minimize the energy consumption of the device [45]. However, the light sensors of smart wearables can also be used to measure a person’s general lighting conditions and adjust controllable light sources (e.g., [46]). In general, the light sensors of smart wearables cannot detect the ambient color temperature or ambient colors. In some use cases, such as the assistance of visually impaired people, special light-sensing devices are used for this purpose [47]. However, modern smartphone cameras can be used as well. Based on the brightness and color of the display or other reference object, a smartphone camera may be able to detect the color and texture of surfaces, liquids, or objects with good precision (e.g., [48,49]).

### 2.3. Factors with Affective Effects

The PLE can have a direct and indirect effect on learners via mediators. Such mediators include learners’ emotional states, moods, or motivations. Learners’ cognitive performances and willingness to engage can be affected by these mediators (e.g., [50]). We identified several factors in the literature that may influence these mediators. These factors are spatial comfort, presence of others, and self-care.

#### 2.3.1. Spatial Comfort

Learners may perceive some factors of the space in which their learning occurs as positive or negative with regard to their spatial comfort. The effects of air quality in terms of oxygen level and ambient temperature as factors affecting the blood oxygen level are physiological but should also be considered in terms of their influence on learners’ moods. Among other things, the perception of air quality is also subjectively influenced by the smell of objects and people’s body odor. Smell has been shown to influence both human mood and cognition. For example, essential oils of lavender and rosemary, when inhaled, can elevate or maintain mood and increase contentment [51]. Similarly, a comfortable ambient temperature can make a room seem pleasant and thus lift the mood. In addition, the influence of the color temperature and luminance of a room’s lighting can not only be counted as a direct influence on cognitive performance, but could also be driven by the affective mood changes of learners (e.g., [40,41]). The more positive the learners’ moods, the more willing they are to invest cognitive resources into learning [52]. Ambient noise can likewise indirectly affect cognitive performance by decreasing motivation and thus lowering learning performance [53]. Not only temporary but also permanent features of a space, such as the organization and quality of the facilities as well as the equipment therein, can influence learners’ ease when completing and thus their willingness to engage more or less intensively with a learning task [9].

Air quality could be defined, for example, by measuring odor and smell within a physical environment. Measuring odor and smell is technically feasible but rather complex. Odors and smells are composed of different chemical molecules. A variety of specialized sensors are typically combined to detect such molecules, such as metal oxide, electrochemical gas, optical, surface, and acoustic wave sensors. Such a variety of sensors allows the distinction of the different types of volatile organic compounds necessary, for example, to detect coffee [54], essential oils [55], or fungi [56]. The other factors of spatial comfort we identified, such as ambient temperature, lighting, and noise, are reasonably measurable. Nevertheless, different people may perceive these factors differently depending on their preferences. To determine the perceived spatial comfort of a person, it is necessary to combine the objective sensor data with subjective self-reported experience data from that person. Spatial comfort is perceived very subjectively, so self-reports are necessary and beneficial to measure the required data.

#### 2.3.2. Presence of Others

The presence of a peer learner during learning, or even just the illusion of it, can trigger motivation and facilitate cognitive processes [57]. Thus, learners may spend more time learning and be more engaged in learning activities in the presence of others, so long as they do not contribute to detrimental factors such as excessive auditory noise.

A rough estimate of the presence of others could be possible to obtain by detecting smartphones in the proximity of a person. For example, Bluetooth and Wi-Fi technologies can be used for this purpose [58]. However, smartphones of the newer generations do not necessarily respond automatically to Bluetooth requests, which is a problem. Nevertheless, a specific implementation of human presence detection has been implemented, for example, as a Bluetooth-based contact tracking functionality in some implementations of COVID-19 apps [59].

#### 2.3.3. Self-Care

Not only external conditions but also learners’ self-care can have an impact on learning. In this regard, clothing represents a symbolic means that can have an effect on a learner’s cognitive performance [60]. Therefore, it may be reasonable for students learning from home to prepare themselves for learning sessions as they might for an in-person event or in the workplace. Such preparations could range from body hygiene measures such as brushing teeth and combing hair to dressing in work clothes and stretching exercises.

Measuring an individual’s self-care is most effectively achieved through self-reported data. With the help of 3D cameras it is possible, for example, to detect clothing changes [61], but the fittingness cannot be evaluated in this way. To reduce subjectivity, it may be beneficial to provide a guideline or offer the person the opportunity to receive an independent second opinion.

## 3. The Software Infrastructure

After identifying factors from the PLE that might affect learning and researching instruments that can be utilized to measure these factors, the next task this research addresses is collecting the desired data.

### 3.1. Requirements and Considerations

Before the initial architecture design was created, some design decisions have to be made by the software architects. These decisions include device selection, method selection, session flow, orchestration, and data format.

#### 3.1.1. Device Selection

The literature search in the previous section (Section 2) shows that smart wearables such as smartwatches and smartphones have been used in research to measure factors from the PLE. Smart wearables provide a variety of sensors such as light sensors, microphones, temperature sensors, heart rate sensors, blood oxygen saturation sensors, accelerators, gyroscopes, GPS, Wi-Fi, Bluetooth and NFC. The literature also shows that smart wearables are suitable devices to collect self-reported data using ecological momentary assessment (EMA) (e.g., [62,63]). Self-reported data are often necessary to capture those factors that are not simple to measure with sensors, such as the presence of others, or enhance the combination with self-reports such as spatial comfort. This is particularly true for factors related to learners’ affective states.

#### 3.1.2. Method Selection

We chose mobile sensing as the method for collecting the PLE factors. This method allows us to collect two types of data. First, we can collect sensor data from the devices transparently to the student. Second, we can gain additional information and confirm sensor readings through self-reports on the smartwatch. Self-reports are queried using questionnaires or micro-questionnaires that are displayed on the smartwatch at configurable times. Questionnaires typically consist of multiple questions with a range of response options. These questionnaires are used to ask about multiple permanent factors such as semantic location. On the other hand, micro-questionnaires are used in learning sessions to capture temporary factors such as perceived noise level. By these means, we aim to minimize the sampling burden for students within their learning sessions by utilizing micro-interactions [62].

#### 3.1.3. Session Flow

Certain restrictions apply to the use of smart wearables for data collection, such as limits on battery consumption, processing power, and network bandwidth usage. We intended for users to have direct control over the timing and duration of data collection for self-determination and privacy reasons. For this reason, users need to be instructed to open the application on their own and start a session whenever they intend to begin learning. Sensor data, as well as EMA data, were collected only within a session, according to the design. This restriction to learning sessions reduces the consumption of device resources by the application. Not limiting data collection to learning sessions but collecting it throughout the day could provide a more holistic perspective on a learner’s activities but it turned out to be much more complex for a generic software design and more invasive for the users. The design also calls for cyclically reminding users that data collection is still active and should be stopped when they ended their learning. However, it is up to the users to decide when to stop the sessions.

#### 3.1.4. Orchestration

Data collection in mobile sensing needs to be orchestrated during the execution of a session. For Edutex, the orchestration takes place continuously on the server-side part of the infrastructure. Events that trigger self-report and sensor data collection are sent by an orchestrator module. This type of orchestration allows for a more flexible intervention model in future iterations of the project. A unique feature of this approach is that a near-continuous connection to the device must be maintained. To tolerate disconnections, the connection channel of the software infrastructure is set up to tolerate connection losses of up to 30 min without dropping the session. If the connection is re-established in the meantime, the session will continue seamlessly and without disruption to the user. After the timeout, the session is automatically terminated, and the user must start a new session.

A WebSocket connection is used for communication between the client and the server. Through this connection, the client is currently given instructions and the collected data are sent back to the server. This type of connection is also used by other applications such as email so clients can maintain a constant connection and quickly exchange new events. The study described later in this paper did not include interventions based on the collected data. However, interventions based on data from multiple data sources, including learning management systems, are planned in subsequent iterations of this project. We have opted for the ability to push interventions rather than have the client ask for them. Maintaining a WebSocket connection between the client and server is, we believe, more timely for just-in-time interventions triggered by an analytics service from the server. The stream of events arriving at the client is usually very sparse, as typically only a few short commands are sent. With an HTTP API, smartphone clients would need to perform continuous fast polling unnecessarily. However, the amount of data streamed from the client side to the server side depends on the study design.

**Figure 3:**
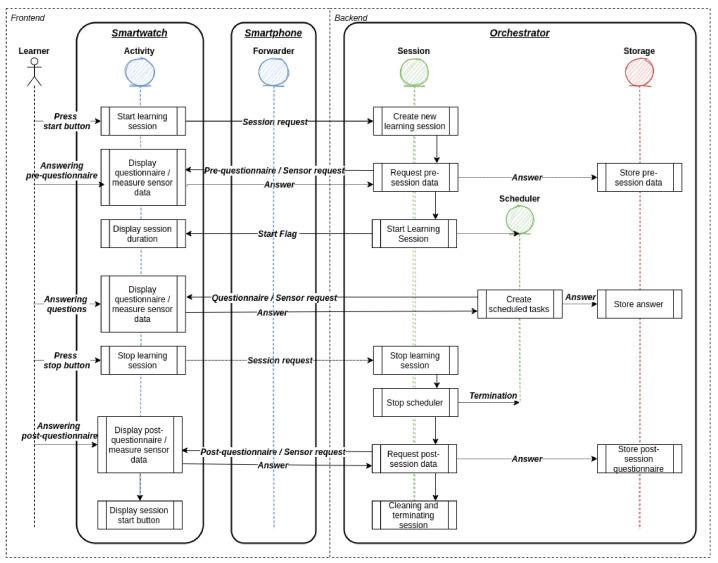
This figure displays the data orchestration of Edutex in the exploratory mode. Study designers can collect sensor and self-reported data before, within, and after the student started a learning session.

The infrastructure provides data in two modes. These are the exploratory mode and the intervention mode. The orchestration is visualized in Figure 3. The exploratory mode allows data to be collected in snapshots. With a snapshot, the momentary sensor measurements are captured. A snapshot can be configured in the administrative interface of the server side to set up a variety of sensor hooks. Each sensor hook is configured at the time of activation to collect data in the specified period and with the specified accuracy. At the end of each snapshot, the collected data are transmitted to the server side in the specified data format. This mode is intended for exploratory studies where data are collected for data analysis purposes. The intervention mode allows sensor data to be collected in a continuous stream. In this mode, the sensor hooks are configured to continuously collect sensor data and relay them as they become available. Smart wearable operating systems typically limit the fidelity of sensor data collection to reduce the resource consumption of the devices. The Android operating system unofficially limits the fidelity to 100 Hz. If the study design requests many data from sensors with high resolutions, bandwidth usage can become high. Therefore, study designers must be made aware of this before configuring sensor hooks. Collecting data in this mode with a near-real-time data stream can be used, for example, to facilitate stream processing or apply AI models. The results could be used to make predictions or detect specific conditions that could, in turn, trigger just-in-time interventions.

#### 3.1.5. Data Format

**Figure 4:**
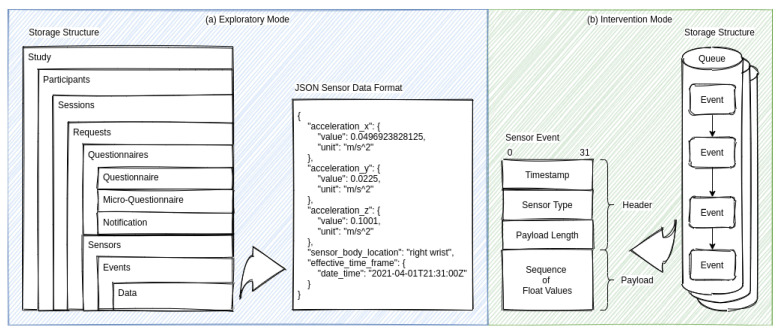
This figure displays the storage structures and data formats used in the two data collection modes. The data schema used in exploratory mode (**a**) is either JSON specified in the OpenMobileHealth project or aligned with it. The data schema used in the intervention mode (**b**) is transmitted without a schema to reduce size.

A suitable data format is beneficial to ensure the comprehensibility of the data. In addition, using an established data schema can reduce immaturity and promote data re-usability. Due to the different requirements that the two supported modes of the infrastructure have, different data formats were used. The left box (a) in Figure 4 shows the database storage structure and the detailed JSON sensor data format used in the exploratory mode. The right box (b) in Figure 4 shows the message broker storage structure and the detailed sensor event format used in the intervention mode.

For the exploratory mode, we decided to use the Open Mobile Health (OMH) data format whenever possible and appropriate (https://www.openmhealth.org, last accessed on 5 August 2021). The OMH standard provides an excellent schema and facilitates uniformity. Some of the sensor data that the infrastructure collects are not yet specified by the OMH standard. This is mainly because the OMH standard is predominantly oriented to physiological characteristics. Therefore, we specified similar data schemas for unspecified sensor types such as ambient noise and illumination levels. Since we were not aware of a suitable standard for EMA data, we derived our data format based on the OMH data schemas. The storage structure is oriented from the perspective of a study designer. Here, the study designer can relate EMAs to sensor data directly during the study design process in the administration interface.

The selected data format should not consume an unnecessary amount in the intervention mode. Therefore, the transmitted data are reduced by any schema in this mode. Each event contains only the collected data values in a well-defined sequence. This approach allows the prototype and related projects to transfer larger amounts of data with high throughput when needed. In the implementation, the software infrastructure uses the Apache Avro (http://avro.apache.org, last accessed on 5 August 2021) system for storage and communication in the Event Store for this purpose. This system allows managing the data schema separately from the data utilizing a schema registry.

### 3.2. Software Architecture Design

**Figure 5:**
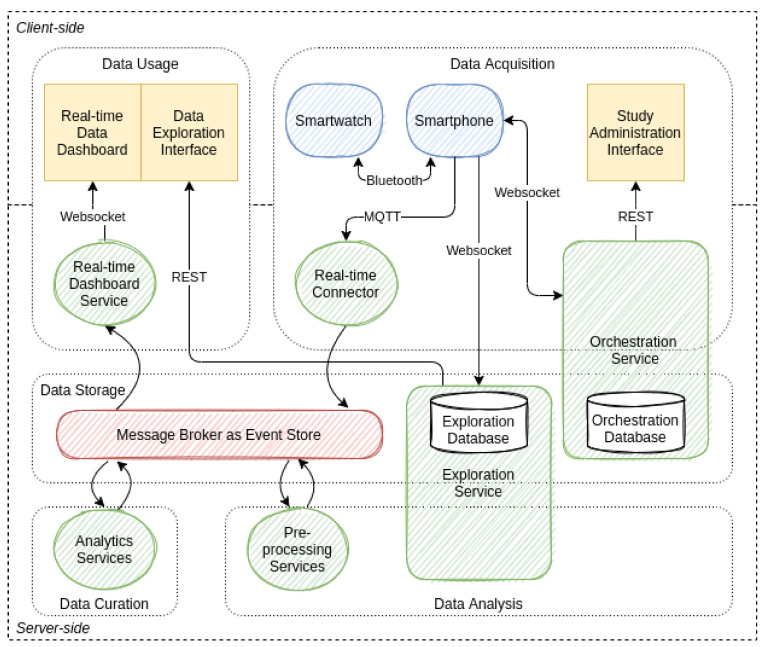
This figure shows the software architecture design of Edutex. The design differentiates between the client-side part and the server-side part. The client side comprises modules for data acquisition and data usage. The server side encapsulates the processing logic for data acquisition and data usage as well as data curation, data analysis, and storage.

For the software architecture design, we used the Big Data Value Chain (BDVC) [64] as a guideline. The BDVC can be used to model the high-level activities that make up any information system [1]. The BDVC identifies the following key high-level activities: data acquisition, data analysis, data curation, data storage, and data usage. Corresponding to these five components are the learning activity sensors, data source connectors, orchestration service, preprocessing service, analytics service, message broker, and dashboard service. Figure 5 illustrates this.

#### 3.2.1. Learning Activity Sensors

The learning activity sensors are the software units that measure the data about the factors of the PLE and the learners at the data sources. The hardware sensors of the smart wearables and the self-reports of the users are the data sources we currently measure with the application.

#### 3.2.2. Data Source Connectors

The data source connectors provide the interfaces for data exchange between the server side and client side. The protocols WebSocket, REST, and MQTT are used for this purpose.

#### 3.2.3. Orchestration Service

The orchestration service provides an administrative interface to configure study designs, including the data inputs. The study designs are composed of questions and answer choices as well as the assigned sensor requests. Furthermore, questions can be aggregated to questionnaires and micro-questionnaires. Studies encapsulate these types of surveys and are assigned internally to registered participants. The participants click on the session start button on the smartwatch starts a new session process in the orchestration service. During the session, the questions and sensor requests are orchestrated live. Administrators can also review the live data stream for troubleshooting. After a session is completed or a study is finished, the administrators can also export all the acquired data from this service.

#### 3.2.4. Message Broker

The Message Broker is primarily used for data transfer between the different components of the infrastructure. Its second task is to permanently keep the communicated events in their original sequence. This also makes the message broker an event store. The data are organized within the event store in so-called topics. A topic is created for each data source, such as a specific sensor on a specific smartwatch. The session IDs serve as keys. The retention of the raw data enables reprocessing at a later point in time and the traceability of calculations.

#### 3.2.5. Preprocessing Service

The preprocessing services enable the incoming data stream to be cleaned, filtered, transformed, and the required features to be calculated. The output of this data stream serves as input for the analytics service. On the one hand, the data must be preprocessed so that AI models can make just-in-time predictions. On the other hand, the preprocessed data are needed as training data for the new AI models after a review step by the learning expert. The current version of this service only performs basic aggregations and filters the data streams.

#### 3.2.6. Analytics Service

This is where previously computed and stored AI models are loaded and executed. At runtime, these services register for the preprocessed data streams of the preprocessing services. After the various indicators have been calculated, the services publish their results again in their dedicated topics. These topics can then in turn serve as input for further analysis services or data usage.

#### 3.2.7. Dashboard Service

The Dashboard service is a first attempt to use the resulting data streams by registering for the output topics of the LA service and reading the indicator results. In the first version, the data are only restructured in a rudimentary manner and made available to an HTML5 dashboard via the WebSocket protocol. The dashboard allows an instructor to look at aggregated data streams of online recordings from participants with a number of technical statistics.

#### 3.2.8. Privacy and Data Protection

As responsible researchers and developers, we need to think about privacy and data security early in the design process. This allows us to provide a trusted and secure experience for study participants. As researchers and developers in Europe, this also makes us compliant with GDPR [65], particularly with the regulations “Data protection by design” and “Data protection by default”, as formulated by Article 25 of the GDPR.

In terms of data protection, the prototype enables study organizers to assign each participant a unique pseudonym at the beginning of a study. Thus, the data of the participants can no longer be directly associated with their identity. Furthermore, we only collect technical identifiers of devices. All sensor information that could re-identify individuals is also hashed with a secret salt in the user-side application using the SHA-1 (https://nvlpubs.nist.gov/nistpubs/FIPS/NIST.FIPS.180-4.pdf, last accessed on 5 August 2021) algorithm. This refers to WIFI and Bluetooth BSSIDs. The GPS coordinates are fuzzed in the client-side application using the geohash (https://mmcloughlin.com/posts/geohash-assembly, last accessed on 5 August 2021) method. With a geohash length of 6, we consequently distort the coordinates by 610 m. Thus, an exact position determination on the server side was no longer possible (implications are discussed in Section 6).

The Android operating system, operating the smart wearables used in the field study, is developed by the company Google (https://www.google.com/, last accessed on 5 August 2021). In the prototype, we wanted to prevent cloud services from Google from possibly analyzing participant data. For this reason and performance reasons, we used a direct channel between the smartwatch and smartphone. Therefore, we used the ChannelClient API instead of the DataClient API or the MessageClient API (https://developer.android.com/training/wearables/data-layer, last accessed on 5 August 2021). In the DataClient API, the data were synchronized via Google Cloud Services in some use cases. The ChannelClient API, on the other hand, provides a direct channel between the two devices. However, all of the mentioned APIs are proprietary implementations of Google.

To ensure data security, all communication between the user-side application and the server-side application is encrypted. This refers to the MQTT, HTTP, and WebSocket protocols used.

In the software infrastructure, a selection of the rights of the data subjects as defined by chapter 3 of the GDPR was automated as an independent user-specific feature. This allows all users to log into the web interface with their credentials and execute their rights independently. In particular, the web interface enables participants to download their data as a JSON-specified file as part of their right of access (Article 15) and right of data portability (Article 20). Furthermore, participants can delete all of their data as part of their right to erasure (Article 17). Since it is complex to implement a subsequent correction of data entries in the context of an automated feature, participants can delete the data of individual sessions. This allows them to exercise their right to rectification (Article 16) simply and autonomously, whereby study operators lose a considerable amount of data. Finally, the right to rectification (Article 18) has been automated. This restriction prevents study operators from exporting the data of these participants before any conflicts or misunderstandings have been resolved and the participants have released the data usage.

## 4. Method

A small pilot study with ten participants was conducted by us to properly evaluate the software design and implemented application.

### 4.1. Participants

The study was conducted from December 2020 to March 2021. Due to pandemic restrictions, we were only able to recruit colleagues and associate students. Before the participants could start the study, they were informed in writing about the procedure and its purpose. In addition, all collected data were explained, and the subsequent use was described. This procedure ensured informed consent to the study. In addition, no personal data were collected (see Section 3.2.8). A total of ten participants participanted. Of these ten participants, seven were men and three were women. Participants were between 20 and 45 years old and spanned educational levels from undergraduate, to graduate, and post-doctoral levels.

### 4.2. Study Design

Preconfigured smartwatches and smartphones were provided to all participants for the study. When the devices were handed over, participants were shown how to use the application and the devices and instructed on how to participate in the study. Participants were expected to use the application over several days and in several sessions at different times of the day. If possible, they should use the application at different learning locations. However, this was not always possible due to COVID-19 restrictions.

We divided the study design into five phases: pre-survey, pre-session, in-session, post-session and post-survey. The session phases can be repeated at will. In the pre-survey and post-survey phases, participants each completed a questionnaire in an online tool (https://www.soscisurvey.de/, last accessed on 5 August 2021). Each session also started and ended with a few questions and sensor sampling. However, the questions were asked directly after the participant’s interaction on the smartwatch. In the in-session phase, the questions and sensor queries were asked and retrieved at intervals.

#### 4.2.1. Pre-Survey

Before the participants launched the first session, they completed a questionnaire about their demographics and affinity to technology in an online survey tool.

#### 4.2.2. Pre-Session

At the beginning of each session, the participants reported permanent factors of the learning environment using a questionnaire prompt on the smartwatch and recorded simultaneously using the appropriate sensors. Questions were asked about the semantic location and the spatial comfort such as “At which location are you studying at the moment? Options: Home office, work office, café, library, outdoors, other location”. The associated sensors were GPS, Wi-Fi, and Bluetooth.

#### 4.2.3. In-Session

During the sessions, sensor data were recorded approximately every 20 min. Simultaneously, the application probed participants situational factors from the physical environment such as “I feel comfortable with the temperature and humidity of my current study room”. The sensors used included the microphone, heart rate, accelerometer, and light sensor. In parallel, the application sent the sensor data in a continuous stream during the ongoing session on the sensor data stream channel.

#### 4.2.4. Post-Session

After participants indicated completion of a learning session by pressing the stop button on the Smartwatch display, the application displayed a final questionnaire. The final questionnaire was used to test the usability of complex standardized questionnaires in the application context. To ensure that participants did not forget to stop the application once they had stopped learning, they were sent a notification every 60 min. In this notification, they were informed that the application was still active.

#### 4.2.5. Post-Survey

At the end of the study, participants completed a final questionnaire that included questions about usability such as “How comfortable were you with the navigation within the questionnaires?” and about their experience with the application such as “How would you rate your experience with the application?”. In addition, questions were asked about the perceived invasiveness of the data collected, such as “How invasive do you consider the sensors that have been used to collect data about you?”, and motivation to reuse the application, such as “How motivated are you to use this application again in another study?”. Furthermore, open-ended questions were asked about desired feedback and missing features such as “What improvements would you make to this application to make it better for you and others?” and “If you have any additional comments or feedback about the application, please share them here”.

### 4.3. Compensation

There was no financial compensation in this study, neither for participation nor for achieving high compliance. Participants were only offered the opportunity to try a regular smartwatch for the duration of the study.

### 4.4. Implementation

**Figure 6:**
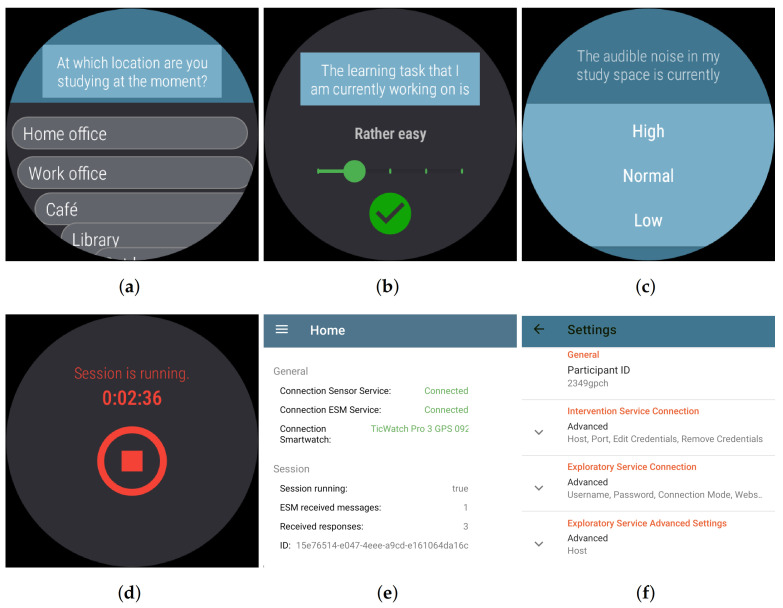
Figures (**a**–**c**) show different display and input formats for questions on the smartwatch: (**a**) a vertically scrollable list of answers allows to offer a wider range of options; (**b**) a horizontal slider enables participants to give their ratings on a scale; and (**c**) one click on an answer option in a micro questionnaire and it vanishes. The session duration is displayed in the interface shown in figure (**d**) when the session is running and no response is requested. Figures (**e**,**f**) show part of the smartphone interface: (**e**) this overview helps participants to see the status of the application; and (**f**) in the settings they can set up the server connection and credentials.

For the development and evaluation of the prototype, we looked for an operating system and devices that are favorable to an open-source project. The decision fell on Android devices (https://www.android.com/, last accessed on 5 August 2021). In our opinion, development for this operating system is very developer-friendly and future-proof, and no special development environments are needed. Android devices also have good market coverage and are relatively inexpensive. In particular, we selected Mobvoi TicWatch Pro 3 GPS (https://www.mobvoi.com/ge/pages/ticwatchpro3gps, last accessed on 5 August 2021) and Google Pixel 4 (https://support.google.com/pixelphone/answer/7158570, last accessed on 5 August 2021) devices. The application must be installed on both paired devices for use in studies. The primary participant interaction took place on the smartwatch. Only server-side communication and authentication settings were altered by the participant on the smartphone. Furthermore, the smartphone interface displays rudimentary status information. Figure 6 shows snapshots of the client interfaces.

## 5. Results

The ten participants successfully recorded themselves in a total of 55 learning sessions. We cleaned the data before analysis by filtering out sessions that were canceled due to technical errors. The sessions lasted on average 78.07 min and were about equally distributed over the morning (28) and the afternoon (27). The application performed 620 sensor queries during these sessions, with the sensors returning in total 892,938 individual sensor events. To collect self-reports, a total of 174 questionnaires were displayed to the 10 participants. On average, they had to be prompted 1.22 times to answer them. When answering the questionnaires, 98.9% of the questions were answered successfully. Whether some questions were skipped intentionally or by mistake could not be determined technically. It took the participants about 46.6 s to answer the questionnaires. These results are summarized in Table 1.

### 5.1. Data Quality and Expressiveness

**Figure 7:**
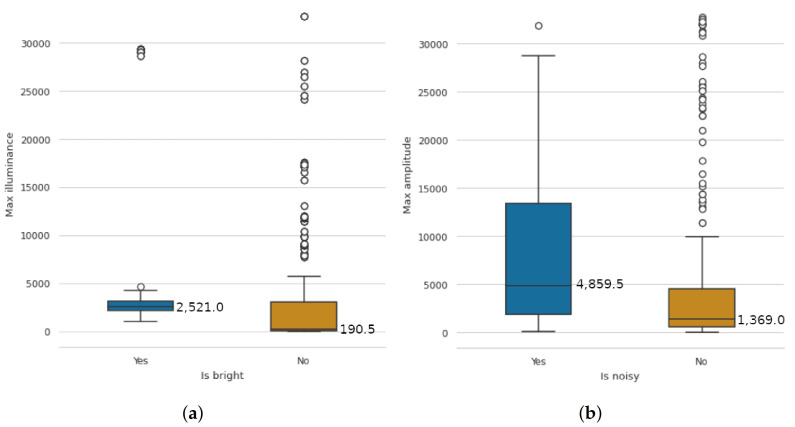
This figure shows the sensor data related to the two PLE factors: (**a**) lighting: The data show that, on average, the lighting conditions measured by the sensors match the self-reported PLE conditions. The median of the not very bright environment is 190.5 lx, which corresponds to normal room lighting. The median of the very bright environment is 2521.0 lx, which corresponds to very bright room lighting; and (**b**) audible noise: The data show that, on average, the noise conditions measured by the sensors are consistent with the self-reported PLE conditions. The median amplitude of a not very noisy environment is 1369.0 (62.7 dB), which corresponds to a regular noisy background. The median amplitude of a very noisy environment is 4859.5 (73.7 dB), which corresponds to a very noisy background.

The recorded sensor data of the ten participants show an expected level of interference in the evaluation. There were some measured values for the light sensor and the microphone that we classified as outliers after a visual analysis. For this reason, we did not calculate the mean values for the analysis but the median and quartiles. The mean value is prone to outliers, as demonstrated by the high standard deviation, making the median a more robust measure for our use case. In the study, we asked participants if they perceived their environment as very bright and if they perceived their environment as very noisy at the current time. We were able to read the lighting conditions and the noisiness of the PLE well from the sensor data with regard to the self-reports of the participants. Thus, bright and non-bright environments can be well distinguished in the data, and the prevailing noisiness can also be well inferred from the measured data. In both cases, the values of the outliers are very scattered and sometimes take on extreme values. These measurements can be caused by clothing that slides over the sensors, such as a sweater sleeve, or by the unidirectional sensors. The measurement results are presented as boxplots in Figure 7.

In most cases, the location could be clearly assigned to the semantic position indicated by EMA. The participants gave different information within one session in three cases, indicating statements made by mistake. As expected, the Wi-Fi identifier could be reassigned to the geoposition in all cases. Thus, an already known study space can be re-identified by Wi-Fi with a high probability. If no Wi-Fi signal is available, a unique assignment is possible, as long as the positions are outside the distortion of 610 m introduced intentionally with the geohash method (see Section 3.2.8). With an available Wi-Fi signal, unambiguous identification is also theoretically possible at a shorter distance at the signal capacity of the Wi-Fi.

The Bluetooth data were very mixed. The number of devices that the sensor had detected varied greatly within even one session. New devices were added again and again within the sessions, and some were no longer detected. Thus, we could not reliably use this data to re-identify a context. In addition, there was no consistent correlation between the number of devices detected and the number of other people in their PLEs reported by the participants.

All further sensors were only measured and collected for the technical evaluation but not further analyzed because our study design did not yet allow for this.

### 5.2. Software Implementation

In the following, we evaluate the implementation according to performance, scalability, extensibility, and versatility. The implementation of the research prototype is made available to the community as an open source (https://gitlab.com/ciordashertel/edutex, last accessed on 5 August 2021).

#### 5.2.1. Performance

The MQTT client is crucial for the evaluation of the client-side application on the client device. In the implementation, we used the Eclipse Paho MQTT client library (https://www.eclipse.org/paho/, last accessed on 5 August 2021). A requirement for the use of mobile sensing was that the client should be able to handle a momentary client-side message pushback. This case can occur when there is a temporary shortage of network bandwidth because the sensors continue to generate events at the same frequency.

The used library provides a queue for up to 65,536 messages for these so-called “in-flight messages”. When the queue is full, any subsequent messages are discarded. This results in maximum memory consumption of 100 bytes × 65,536 = 6400 KB for an average message size of 100 bytes. There is, therefore, no risk of a memory leak in this case.

The further evaluation of the intervention mode of the prototype was based on the assumption that all sensors described in Section 2 must be transmitted in a frequency as high as possible. This refers to the sensors for acceleration, light, audible noise, heart rate, blood oxygen saturation, temperature, Bluetooth, Wi-Fi, and GPS. The sensors for Bluetooth and Wi-Fi can only be retrieved once per minute in the Android operating system and the GPS signal only with a frequency of 1 Hz. The maximum amplitude for volume measurement was only evaluated by us every 5 s. The sampling rate of the remaining five sensors is limited to approximately 100 Hz by the Android operating system. This results in a calculated maximum threshold of (1 + 1 + 60 + 20 + 5 × 6000) events/min = 30,082 events/min = 501.3 events/s. For simplicity, we assumed 500 events per second in our evaluation. Since the individual events are transmitted as individual MQTT messages in the intervention mode, this results in an average load of 500 Hz by each client for the data source connectors.

We tested the throughput of the server-side data source connector using the benchmark tool MQTT-bench. The test was performed at the local host level to eliminate the network throughput factor. We loaded the connector several times with 50 clients, 10,000 events per second, and an event size of 100 bytes. This resulted in an average throughput of 38,684 messages for the Mosquitto MQTT Broker (https://mosquitto.org/, last accessed on 5 August 2021) used, which corresponds to approximately 773 messages per second per client.

However, after the MQTT broker has received the messages, they are written to the message broker via another connector in the implementation. The Kafka MQTT connector used was able to achieve a maximum throughput of 23,780 messages on average, which corresponds to approximately 475 messages per second per client. With a maximum throughput of 500 Hz per client, this results in a maximum limit of approximately 47 participants that can simultaneously send data to one server-side data source connector. Horizontal scaling of the components is necessary to support additional users in parallel. This is supported by the use of container technology (see Section 5.2.2).

We used Kafka technology for our implementation of the message broker. Kafka has a throughput of several 100,000 messages per second (https://www.confluent.io/blog/kafka-fastest-messaging-system/, last accessed on 5 August 2021) per instance. Therefore, we refrained from testing the Kafka brokers.

#### 5.2.2. Scalability

For the deployment of the server-side part of the software infrastructure, we decided to use Docker (https://www.docker.com/, last accessed on 5 August 2021) containerization technology. With this technology, it is possible to run applications independent of operating systems and isolated from other sensitive applications. In addition, this technology offers the possibility to document the configuration and operation transparently and in detail. Since the prototype uses public Docker images for standard components such as MQTT, MQTT-Connector, and Kafka, they are easy to update or replace by other developers. The implementations can also be converted into images by operators using specified Dockerfiles. The use of containers simplifies the scaling of the infrastructure for different requirements. The detailed setup can be reviewed in the source code repository.

#### 5.2.3. Extensibility

The application can be easily extended for expansion by newly available sensors of the smart wearables. For this purpose, the applied pattern can be adopted during software development. The interfaces on the client and server sides were designed generically for this purpose. A software-side extension by new types of EMA is not planned because we have not yet seen any need for this. Nevertheless, the project is open to extensions by the community. The design of the existing implementation can be easily adapted.

#### 5.2.4. Versatility

By enabling the configuration of the measurement instruments and measurement intervals in a server-side administration interface, we consider the versatility of the application to be high. Study organizers can thus create and test various study designs. Furthermore, study designs can be adapted or exchanged without user involvement. This makes it possible to adapt the application in a versatile way for longitudinal studies or adoption.

### 5.3. Qualitative Feedback

At the end of the study, we asked the participants to answer a questionnaire with open questions. Here we gained good insights from the participants about the application. None of the participants in this study had used a similar application before. As was already to be expected due to the different affinity of the users for technology (7 scale points, M 3.6, SD 1.5), the users rated their experiences with the application varied but positive (5 scale points, M 3.3, SD 1.2). They did not perceive the collected sensor data as invasive and responded favorably about using this data for research purposes (5 scale points, M 2.0, SD 1.5). Participants liked using the applications to learn more about the many influencing factors from their PLE. They rated the factors presence of others, lighting, and air quality from their PLEs as the most crucial to their learning productivity. Altogether, they were missing direct feedback from the application. It was suggested that there should be a dashboard with reflection possibilities and recommendations for improvements. For this, it was proposed by one participant that the dashboard should alert learners to likely unnoticed factors in their PLE. Furthermore, gamification aspects were suggested. To this end, it was proposed by another participant that users should be able to self-track themselves in PLE individually optimized learning sessions.

## 6. Discussion

To extend the power of learning analytics in distance learning at home, it is advantageous to additionally include factors from students’ PLE. For this purpose, in line with So- LAR’s definition of LA (https://www.solaresearch.org/about/what-is-learning-analytics/, last accessed on 5 August 2021), we addressed in this paper how we could identify, measure, collect, process, and report these factors using multimodal data sources.

To the best of our knowledge, LA researchers have paid little attention to the effects of the PLE on learners in distance education at home. In experiments, the PLE is usually treated as a constant variable. One goal of this work was to determine what factors from the PLE can have an effect on learning. To answer the first research question (RQ1), we identified different factors from the PLE in the literature. We then grouped these factors into nine types and assigned their effects to the three categories of cognitive effects, physiological effects, and affective effects. These factors, contrary to common belief and student practices, may be counterproductive to learning by exerting unconscious influence, for example, retention loss increases in learning environments such as the cafeteria or during online messaging [66]. The identification of these factors with the help of the relevant literature enabled us to derive requirements and considerations for the design of the software infrastructure and to outline our further procedure.

After identifying factors from the literature, we addressed, in the course of answering RQ2, which instruments we could use to measure these factors in the PLE of students learning at home. Here, our literature search revealed that smart wearables are well suited to be utilized for this purpose. Moreover, we specifically selected smartwatches as devices as they have shown to be a low burden for EMAs in everyday life [62]. Crucial in this regard is that sales of smart wearables, and smartwatches, in particular, are increasing rapidly (https://www.gartner.com/en/newsroom/press-releases/2021-01-11-gartner-forecasts-global-spending-on-wearable-devices-to-total-81-5-billion-in-2021, last accessed on 5 August 2021). Thus, these devices will soon be widely available instruments for students with which their learning process could be monitored and supported. In addition, smartwatches are equipped with an increasing number of sensors. The owners of such devices are primarily focused on the physiological sensors that are being used in the course of the e-health movement. Many specialized sensors that were previously only installed in dedicated devices, such as sweat glucose monitors, are now being actively miniaturized and installed in smart wearables. Nevertheless, smart wearables also have sensors that can measure movement and the physical environment. The information from these sensors primarily serves and improves the user’s interactions with the device. However, the results of our literature review revealed that there are more advanced application areas such as human activity recognition and context recognition. The community could leverage these recognitions to enable sensor-based quantified-self learner applications, for instance.

After identifying the factors and effects and searching for instruments of measurement, we were able to proceed to design the software infrastructure in accordance with RQ3. For the design, it was first necessary to define some requirements and make trade-offs. For the device and method selection, we chose mobile sensing sensors and EMAs on smartwatches and smartphones. By this selection, we expect a higher coverage of end-user devices and thus a higher probability of usage in subsequent studies. However, we are well aware of the fact that there are several providers on the market whose applications are unfortunately not compatible. For development time, price and market coverage reasons, we have chosen Wear OS operating devices. Implementing an exploratory mode and an intervention mode may allow study designers to iteratively develop a research case and finally deploy it in situ. In addition, it became apparent during planning that the exploratory and interventional uses of Edutex require different orchestration and data formats. Whereas in the exploratory phase a flexible UI and human-readable formats matter, in the intervention phase, performance and reliability count. The insights and artifacts as outputs of the exploration are inputs of the intervention. This particularly refers to preprocessing steps where the data are exported and externally analyzed. The insights gained from data mining and the trained AI models are the output of the use of the exploration mode. Such AI models will in later iterations of this project be installed in the intervention mode.

Using the “Data Protection by Design” and “Data Protection by Default” guidelines early in the software design process, we have been able to ensure data privacy and data protection without limiting the use cases. This allows the software infrastructure to meet the needs of study participants and study organizers at the same time. By implementing the Data Subject Rights as an automated feature to be used autonomously, users can control their data independently of study operators. Through this, we not only want to be compliant with GDPR but also hope to enable participant agency. With technology giants such as Google, Microsoft, and Apple providing this functionality, the same should be true for the education infrastructure.

In order to evaluate the extent to which the prototype can provide relevant information about the learning context in practical use as part of answering RQ4, we tested the first stable prototype with 10 participants. We evaluated the prototype by asking participants to conduct multiple sessions in varying contexts. For this, we also instructed participants to choose different, if possible, places and schedules. Through this study, we were able to collect and analyze data from 55 sessions. The evaluation showed that Edutex could be successfully used to predict self-reported contexts based on the collected sensor data. In particular, we successfully predicted the lighting conditions, volume levels, and positions of the learning contexts. This shows that it is potentially possible to detect factors from the PLE without explicit human involvement. Thus, the application can be used to learn more about the physical contexts when students are distance learning. However, the results show that the sensor values can be quite sensitive to interference if they are covered and touched, for instance, or if the space is equipped in unconventional ways. Additionally, the students’ answers can be given incorrectly if they read the questions only superficially because they are perhaps under stress or absent in thought. An accurate statement about conditions should only be made based on a large amount of reliably collected data. For this reason, it is also important that users review their data and rectify them if necessary (see Section 3.2.8). Based on the findings, it would be possible to provide extended feedback or initiate adaptive just-in-time interventions. However, in the conducted study, this was not yet carried out, which was conveyed to the participants at the beginning, but they still remarked on it at the end. The positive feedback from the participants indicates that the client application provides a good user experience to the individual. However, although the prototype was well-rated, participants missed an appropriate representation of the data obtained about their learning context. This extension will be addressed in upcoming interations to close the loop from data collection to feedback. However, the comprehensible presentation of the collected data and the inferences based on them will likely prove to be a very complex task and represent a new cornerstone for this project. In this regard, student feedback needs to be integrated especially in the context of interventions based on potentially ambiguous data. For example, through semi-supervised learning. We also evaluated the design and prototype against the performance criteria of power, scalability, extensibility, and versatility. The evaluation found that the design has the potential to be deployed at the institutional level. Through the use of advanced technologies, the software infrastructure can be deployed on-site to meet institutional privacy requirements. In addition, its flexible design options allow it to be used in a variety of application areas.

Since the implementation of the prototype is publicly available and open-source, interested researchers can run the software infrastructure on their premises. Moreover, this allows the implementation to be extended with new functionalities by different actors in the future and to be made available to the community as well.

### Limitations

The developed software infrastructure and the conducted study have several potentials for improvements. One limitation is that the infrastructure has not yet been tested with a variety of devices. The hardware sensors embedded in commodity devices can vary significantly in quality and thus impact the quality of the data collected. Therefore, in further development steps, it is necessary to perform standardized measurements of the factors with different devices and, if necessary, to store corresponding calibrations. Furthermore, at the time of study planning during the pandemic, we opted for a straightforward questionnaire for the final collection of feedback from study participants. In retrospect, it probably would have been better if we had chosen to conduct online interviews. In such a format, participants are often more detailed in their explanations, and follow-up questions can be asked. Another limitation is that the prototype has not yet been deployed in a broad study with various participants using it simultaneously. Such a deployment may reveal unexpected side effects such as bottlenecks in performance. With a higher heterogeneity of participants, the analyses of the multimodal data would also be more expressive. An additional limitation is that, so far, only project participants have created studies and operated the infrastructure. The productive use by third parties will likely reveal ambiguities and flaws in its use. A final limitation is that the prototype has not yet been used with a more general, diverse, and much larger population in a wide variety of PLEs. With a good study design, such a comprehensive study may provide generalized insights into effects due to students’ physical learning contexts.

## 7. Conclusions and Future Directions

The first research aim of this paper was to investigate what factors of a student’s learning environment can have an effect on learning and which effects those factors cause. The literature search results identified nine factors that can have an effect on a learner’s cognition, physiology, and affect.

Based on this, the second research aim of this paper was to determine what instruments could be used to measure these factors using smart wearables. The search showed that all factors could be measured with smart wearables using inherent sensors and self-reports on the devices.

The third research objective was to design an infrastructure that would be able to measure, collect, and process the necessary multimodal data using commodity smartwatches and smartphones. Based on the design, the software infrastructure Edutex was created. The results show that the infrastructure can be used both in an exploratory mode to collect new research data and in an interventional mode for just-in-time interventions. In this regard, the configurable software infrastructure enables flexible design possibilities for studies. The digital provision of data subject rights as defined by the GDPR enables students to have extended data agency.

The final research aim of this paper was to find out to what extent the prototype can provide relevant information about the learning context in a field study. The results show that the factors lighting, audible noise, and context dependency can be inferred well. The factor blood oxygen saturation can be measured outright. Factors that can have an affective effect, such as spatial comfort, presence of others and self-care and the factor visual noise, can be measured well via self-reports on the smartwatch. The last factor, blood glucose level, can only be measured with upcoming generations of smart wearables.

The primary vision use case for this infrastructure is to support self-directed learning in distance education. In times of a pandemic, it has become even more critical for students to self-organize their learning and their physical learning environments. Instruments such as Edutex could support students in doing so by providing information about habits, behaviors, physiological state, and physical learning environments. In doing so, perhaps it should not always be the student who is the addressee of evidence, but rather the instructors or the educational institution, taking into account security, privacy, and informed consent. Educational institutions are envisioned as the primary operators of this infrastructure. These could support students using this tool as part of their educational missions. However, as an open-source project, it could also be operated by student organizations and businesses, for example. The user base is currently limited to Android devices (see Section 4.4), but this only needs to be the first step with good coverage. During development, care was taken to ensure that the infrastructure is server-independent and relatively easy to use. With this structure, the infrastructure can be operated on the premises of many educational institutions with little effort. The insights gained from the collection of the various factors can potentially be used in a variety of use cases. The next planned use case is to analyze the collected data to make users aware of the physical context in which they regularly work, the physical and affective state they are in while doing so, and how and which factors from their environment they may want to improve. In terms of individuals’ conscious and unconscious behaviors, integration with other data sources such as learning management systems would be interesting to explore to determine what learning goals students set for themselves and when distraction, fatigue, stress, or arousal set in. Meaningful recognition and labeling of states and behaviors could help learn models for recognition and identify facilitating factors. If identification, recognition, or even prediction becomes accurately possible, just-in-time interventions can promote performance, volition, or retention in learning. In this context, it might also be of interest to education providers or social service agencies to find out if and what inadequacies of PLEs are associated with social inequality. Access to an appropriate PLE is likely to be related to wealth and social class. In addition, Edutex could be used as an individual tool as part of a range of interventions to help individuals optimize ergonomics, environment, and other working conditions. In this respect, the user base is not only aimed at learners, but also at those who work from home. Not only the individual, but also stakeholders such as employers, supervisors or trade unions need to be considered. These groups also have an interest in promoting health and safety in the workplace and supporting better working conditions when working from home.

There are several approaches to how this project could be extended in technical terms in the future. A first way to widen the application scope would be to futher evaluate the smart wearable applications on various devices with different hardware sensors, display sizes, and input capabilities. A next step to improve the measurement in future work is to extend the multimodal approach with more data sources such as blood glucose measurement, blood pressure measurement, and skin conductance to identify more factors. Providing standard measurement models for the individual factors such as acoustic noise could further enhance the ease of practical applicability. Study creators could thus already use a pre-assessed interpretation of the raw data. To identify new insights and connections of context with learning and compute standard measurement models, the prototype should be applied and evaluated in larger-scale studies. Upcoming studies include investigations of off-task behavior (e.g., [67]) and self quantification of students in distance education at home. Finally, a data storytelling dashboard on the multimodal data should benefit students and instructors in the superficial analysis of behavior and context across learning (e.g., [68]).

## Figures and Tables

**Table 1:** This table shows descriptive statistics about the 55 learning sessions the ten participants conducted in the evaluation study.

	Total	Total Sessions Duration (min)	Mean Session Duration (min)	Morning Sessions	Afternoon Sessions
**Session**	55	5062.48	78.07	28	27
	**Total**	**Sensor Events**	**Location Events**	**Audible Noise Events**	**Lighting Events**
**Sensor** **Request**	620	892,838	350	1715	39,560
	**Total**	**Mean Prompts**	**Questions**	**Answered Questions** **(Percent)**	**Median** **Submission Time** **(s)**
**EMA**	174	1.22	1465	98.9	46.60

## Data Availability

The repository containing the source code of the Edutex software infrastructure is publicly available here: https://gitlab.com/ciordashertel/edutex. The data of the study are currently not made publicly available for data protection reasons and due to further use in subsequent research projects.
